# *Helicobacter pylori* infection in Iran: demographic, endoscopic and pathological factors

**DOI:** 10.1186/s12876-021-01931-1

**Published:** 2021-09-27

**Authors:** Seyedeh Amineh Hojati, Sara Kokabpeyk, Salma Yaghoubi, Farahnaz Joukar, Mehrnaz Asgharnezhad, Fariborz Mansour-Ghanaei

**Affiliations:** 1 Gastrointestinal and Liver Diseases Research Center, Guilan University of Medical Sciences, Rasht, Iran; 2 Gastrointestinal and Liver Diseases Research Center, GI Cancer Screening and Prevention Research Center and Caspian Digestive Diseases Research Center, Guilan University of Medical Sciences, Rasht, Iran; 3 GI Cancer Screening and Prevention Research Center, Guilan University of Medical Sciences, Rasht, Iran; 4 GI Cancer Screening and Prevention Research Center, Gastrointestinal and Liver Diseases Research Center and Caspian Digestive Diseases Research Center, Guilan University of Medical Sciences, Razi Hospital, Sardar-Jangle Ave, Rasht, Iran

**Keywords:** *Helicobacter pylori*, Endoscopy, Pathology

## Abstract

**Background:**

*Helicobacter pylori *(*H. pylori*) infection is the most important risk factor for gastritis and peptic ulcer. However, factors other than *H. pylori* are involved in its pathogenesis. In the current study, we aimed to compare the clinical manifestations and endoscopic and histopathological findings of patients with and without *H. pylori* infection.

**Methods:**

In this cross-sectional study, 233 patients with dyspepsia, referred for endoscopy, were examined regarding the presence of *H. pylori* infection. During an *endoscopic exam*, *5 biopsy* specimens *were taken* from the *stomach*. The criteria for the presence of *H. pylori* infection was the presence and identification of bacteria in pathology. Two groups of *H. pylori*-positive and *H. pylori*-negative patients were compared regarding their demographic, endoscopic, and pathological findings.

**Results:**

Of 233 patients, 154 (66.1%) were non-smokers, 201 (86.3%) were not alcohol users, and 153 (65.7%) used tap water. The most common symptom, reported in 157 (67.4%) patients, was epigastric pain. There was a significant difference between patients with and without *H. pylori* infection in terms of the educational status, occupational status, family history of gastrointestinal cancer, and some gastrointestinal symptoms. Also, there was a significant relationship between the endoscopic and pathological findings of patients with *H. pylori*.

**Conclusions:**

The results of the present study revealed that *H. pylori* infection was not associated with sex, alcohol consumption, or non-steroidal anti-inflammatory drug use. The role of *H. pylori* in the pathophysiology of peptic ulcer was clarified. Also, there was a significant difference in the endoscopic and pathological findings of patients with *H. pylori*.

## Introduction

*Helicobacter pylori* (*H. pylori*) is a gram-negative bacillus and one of the most common gastrointestinal pathogens globally [1]. *Helicobacter pylori* causes chronic gastritis, peptic ulcer, gastric marginal zone lymphoma of mucosa-associated lymphoid tissue (MALT lymphoma), and gastric carcinoma [2]. Recent investigations have reported that *H. pylori* infection is significantly associated with extra intestinal complications, such as iron-refractory iron deficiency anemia (IRIDA) [3], immune thrombocytopenic purpura [4], and vitamin B12 deficiency [5].

*Helicobacter pylori* infection occurs worldwide, although its exact incidence rate remains unknown. Besides, the prevalence of this infection varies by geographic region [1]. Many previous studies have indicated that the rate and risk of infection are significantly high in developing countries with a low socioeconomic status [6]. In this regard, a systematic review by Ghotaslou et al. [7] showed that about 4.4 billion people were infected with *H. pylori* worldwide. Northern America (37.1%) and Australia (24.4%) reported the lowest prevalence rates, while Africa (79.1%), Latin America (63.4%), and Asia (54.7%) reported the highest incidence rates of infection [7].

Contaminated food and fecal–oral routes are the main routes of transmission; therefore, developments in hygiene practices are essential for reducing the rate of infection. Cats, cockroaches, rhesus monkeys, pig-tailed monkeys, and sheep are the main reservoir hosts for *H. pylori* [8]. Generally, patients with *H. pylori* infection are prone to gastric ulcer, gastric atrophy, intestinal metaplasia, and gastric carcinoma [9]. In 1994, this bacterium was recognized as the main carcinogen for gastric cancer [10]. Although the role of *H. pylori* in non-ulcer dyspepsia is unclear, the rate of infection is high in these patients.

The genetic predisposition of the host is very important in *H. pylori* infection [11]. According to previous studies, more than 90% of duodenal ulcers and 60% of gastric ulcers are associated with *H. pylori* infection [12]; however, this association is not entirely known yet. Some studies suggest that the prevalence of *H. pylori* is much higher than peptic ulcer. Therefore, the question arises as to why not all patients with *H. pylori* infection have peptic ulcer disease. On the other hand, many patients with peptic ulcers are negative for *H. pylori* infection. In this regard, Chu et al. examined patients with peptic ulcers from 1996 to 2002, and the results showed that 30% of the patients were negative for *H. pylori*. Also, in a study by Wei et al. in 2014, 62.3% of the patients were negative for *H. pylori* [13].

Poor health standards, cultural background, diet, oral hygiene, large families, consumption of unhealthy water or contaminated vegetables, and swimming in a river are the main risk factors for *H. pylori* infection [14]. There are several methods for the rapid and accurate diagnosis of *H. pylori* infection. These methods can be divided into two categories of endoscopic and non-endoscopic. Non-endoscopic procedures include histology, rapid urease testing, culture, serology, urea breath test, and molecular techniques [15].

Guilan Province is one of the rainy provinces in north of Iran, which lies along the Caspian Sea. Considering the important role of *H. pylori* in peptic ulcer and other gastrointestinal disorders, besides the possible role of other factors, it is necessary to compare patients with positive and negative results for *H. pylori*. Also, no study has been carried out on the difference in the demographic, endoscopic, and pathological data of patients with and without *H. pylori* infection in Guilan Province in north of Iran. Therefore, this study aimed to assess the above mentioned factors in patients with and without *H. pylori* infection.

## Materials and methods

### Patients

From June 2017 to August 2019, in coordination with the endoscopy department of Caspian Gastroenterology and Liver Clinic, patients with dyspepsia, referred for endoscopy, were examined for *H. pylori* infection. Diagnosis of *H. pylori* infection was established for all patients using pathology and rapid urease test (RUT). Patients were then divided into two groups of *H. pylori*-positive and *H. pylori*-negative. The two groups were compared in terms of their demographic information and their endoscopic and pathological findings. All patients, aged above 12 years, who were referred to the endoscopy department of Caspian Gastroenterology and Liver Clinic, were included in this study. The exclusion criteria were pregnancy, use of an acid inhibitor, and use of anti-*H. pylori* drugs.

### Data collection

For data collection, a questionnaire was used in this study, which included the subject’s first name, last name, age, sex, body mass index (BMI), level of education, number of family members, smoking status, alcohol consumption, occupation, marital status, source of drinking water, *H. pylori* infection, family history of cancer, and gastrointestinal symptoms. The questionnaire was delivered to all patients and completed by the patient or the patient’s companion.

### Specimens

Following local lidocaine 10% sedation, upper gastrointestinal endoscopy was performed via video endoscopy (GIF type V, Olympus Optical Co., Ltd., Tokyo, Japan); an endoscopist reported the results. Biopsy specimens were collected from five different regions of the stomach. Also, for histological examinations, four specimens were taken (one from the fundus, one from the body, and two from the antrum). Moreover, RUT was used for the diagnosis of *H. pylori* infection in a specimen from the antrum.

All specimens were placed in a separate bottle, containing formalin 10% and sent to the pathology laboratory. Various premalignant lesions, including intestinal metaplasia (grade I to III), atrophic gastritis, dysplasia, and *H. pylori* infection, were found after fixation, staining, and microscopic examination; they were then classified based on the Sydney criteria [16, 17]. It should be noted that the pathologist was blinded to the patients’ data, diagnostic index, and endoscopic findings.

### Data analysis

Data were analyzed using chi square test and logistic regression (univariate and multivariate). Results were expressed *as* crude *and* adjusted odds ratios (*OR*s) with 95% confidence intervals (*CI*s). All statistical analyses were conducted using the statistics program in SPSS version 21 (SPSS Inc., Chicago, IL, USA). Graphs were also drawn using Prism 7. *P* values less than 0.05 were considered statistically significant.

## Results

The patients’ demographic information is presented in Table 1. The most commonly reported gastrointestinal symptom was epigastric pain, reported in 157 (67.4%) patients. There was a significant difference between patients with and without *H. pylori* infection in terms of the educational status, occupational status, family history of gastrointestinal cancer, and some gastrointestinal symptoms (*P* < 0.001) (Table 2).

**Table 1:** The results of demographic and clinical information of patients

Demographic and clinical data	Frequency (%)
Sex	
Men	125 (53.6)
Women	108(46.4)
Age	
< 35	57 (24.5)
35–45	42 (18.0)
> 45	134 (57.5)
BMI	
< 18.5	3 (1.3)
18.5–24.9	35 (15.0)
25–29.9	145 (62.2)
> 30	50 (21.5)
Number of family members	
< 3	93 (39.9)
$\geqq 3$	140 (60.1)
Smoking	
Yes	79 (33.9)
No	154 (66.1)
Drink alcohol	
Yes	32 (13.7)
No	201 (86.3)
Education	
Illiterate	10 (4.3)
Under diploma	47 (20.2)
Diploma	83 (35.6)
Upper graduated	93 (39.9)
Marital status	
Single	44 (18.9)
Married	186 (79.8)
Divorces	3 (1.3)
Occupation	
Unemployed	56 (24.0)
Housekeeping	71 (30.5)
Employee	39 (16.7)
Self employed	67 (28.8)
Source of drinking water	
River	1 (0.4)
Tap	153 (65.7)
Mineral	48 (20.6)
Tap and Mineral	31 (13.3)
Family history of cancer	
Yes	44 (18.9)
No	189 (81.9)
Gastrointestinal sign and symptoms	
Epigastric pain	157 (67.4)
Heartburn	107 (45.9)
Reflux	76 (32.6)
Weight loss	55 (23.6)
Decreased appetite	64 (27.5)
Early satiety	17 (7.3)
The presence of blood in the stool	9 (3.9)
Iron deficiency anemia	27 (11.6)

*BMI* body mass index

**Table 2:** Univariate and multivariate logistic regression analysis of factors associated with *H. pylori* positive and negative

Demographic and clinical data	Total N = 233 n (%)	*H. pylori* positive N = 109 n (%)	*H. pylori* negative N = 124 n (%)	OR crude (95% CI)	*P *Value	OR adjusted (95% CI)	*P *value
Gender							
Men	125 (53.6)	55 (44.0)	70 (56.0)	0.78 (0.46–1.31)	0.430	0.51 (0.20–1.33)	0.174
Women	108 (46.4)	54 (50.0)	54 (50.0)	Ref		Ref	
Age group							
< 35	57 (24.5)	21 (36.8)	36 (63.2)	1.25 (0.80–2.87)	0.196	0.72 (0.20–2.54)	0.612
35–45	42 (18.0)	25 (59.5)	17 (40.5)	0.60 (0.29–1.21)	0.159	2.47 (0.81–7.53)	0.111
> 45	134 (57.5)	63 (47.0)	71 (53.0)	Ref	–	Ref	–
BMI							
< 18.5	3 (1.3)	0 (0.0)	3 (100.0)	N/A	–	N/A	–
18.5–24.9	35 (15.0)	17 (48.6)	18 (51.4)	1.52 (0.57–4.07)	0.398	1.76 (0.44–7.00)	0.420
25–29.9	145 (62.2)	69 (47.6)	76 (52.4)	1.32 (0.57–3.04)	0.504	1.82 (0.61–5.35)	0.277
> 30	50 (21.5)	23 (46.0)	27 (54.0)	Ref	–	Ref	-
Number of family member							
< 3	93 (39.9)	37 (39.8)	56 (60.2)	0.62 (0.36–1.06)	0.084	0.74 (0.35–1.56)	0.428
> 3	140 (60.1)	72 (51.4)	68 (48.6)	Ref		Ref	–
Smoking							
Yes	79 (33.9)	33 (41.8)	46 (58.2)	0.73 (0.42–1.27)	0.332	0.68 (0.29–1.62)	0.395
No	154 (66.1)	76 (49.4)	78 (50.6)	Ref		Ref	–
Alcohol drink							
Yes	32 (13.7)	16 (50.0)	16 (50.0)	1.16 (0.55–2.45)	0.707	1.75 (0.60–5.08)	0.301
No	201 (86.3)	93 (46.3)	108 (53.7)	Ref		Ref	–
Education							
Illiterate	10 (4.3)	3 (30.0)	7 (70.0)	0.45 (0.11–1.87)	0.277	0.13 (0.02–0.84)	0.033
Under diploma	47 (20.2)	24 (51.1)	23 (48.9)	1.11 (0.55–2.24)	0.765	0.43(0.12–1.58)	0.209
Diploma	83 (35.6)	37 (44.6)	46 (55.4)	0.85 (0.47–1.55)	0.613	0.47 (0.17–1.28)	0.141
Upper graduated	93 (39.9)	45 (48.4)	48 (51.6)	Ref	–	Ref	–
Marital status							
Single	44 (18.9)	12 (27.3)	32 (72.7)	0.35 (0.17–0.73)	0.01	0.38 (0.12–1.13)	0.082
Married	189 (81.1)	97 (51.1)	92 (48.9)	Ref		Ref	–
Occupation							
Unemployed	56 (24.0)	32 (57.1)	24 (42.9)	2.24 (1.08–4.62)	0.029	6.70 (2.22–20.23)	0.001
Housekeeping	71 (30.5)	32 (45.1)	39 (54.9)	1.37 (0.69–2.72)	0.356	1.03 (0.30–3.49)	0.951
Employee	39 (16.7)	20 (51.3)	19 (48.7)	1.76 (0.79–3.93)	0.162	0.90 (0.28–2.80)	0.856
Self employed	67 (28.8)	25 (37.3)	42 (62.7)	Ref	–	Ref	–
Drinking water source							
River	1 (0.4)	1 (100.0)	0 (0.0)	N/A	–	N/A	–
Tap	153 (65.7)	68 (44.4)	85 (55.6)	0.85 (0.39–1.84)	0.688	0.97 (0.36–2.57)	0.957
Mineral	48 (20.6)	25 (52.1)	23 (47.9)	1.15 (0.47–2.86)	0.748	1.07 (0.33–3.46)	0.909
Tap and Mineral	31 (13.3)	15 (48.4)	16 (51.6)	Ref	–	Ref	–
Family history of gastrointestinal cancer							
Yes	44 (18.9)	30 (68.2)	14 (31.8)	2.98 (1.48–5.99)	0.002	3.52 (1.48–8.35)	0.004
No	189 (81.9)	79 (41.8)	110 (58.2)	Ref		Ref	–
Gastrointestinal sign and symptoms^a^							
Epigastric pain	157 (67.4)	85 (54.1)	72 (45.9)	2.70 (1.50- 4.83)	0.001	4.67 (1.92–11.34)	0.001
Heartburn	107 (45.9)	24 (31.6)	52 (68.4)	1.06 (0.63–1.78)	0.895	1.83 (0.89–3.75)	0.009
Reflux	76 (32.6)	34 (44.7)	42 (55.3)	0.88 (0.51–1.53)	0.677	1.35 (0.61–2.99)	0.458
Weight loss	55 (23.6)	34 (61.8)	21 (38.2)	2.22 (1.19- 4.13)	0.013	3.38 (1.34–8.49)	0.009
Decreased appetite	64 (27.5)	35 (54.7)	29 (45.3)	1.54 (0.86–2.76)	0.144	1.62 (0.69–3.78)	0.262
Early satiety	17 (7.3)	8 (47.1)	9 (52.9)	1.01 (0.37–2.72)	0.981	1.73 (0.44–6.77)	0.425
The presence of blood in the stool	9 (3.9)	2 (22.2)	7 (77.8)	0.31 (0.06–1.55)	0.181	0.66 (0.11–3.88)	0.654
Iron deficiency anemia	27 (11.6)	15 (55.6)	12 (44.4)	1.48 (0.66–3.33)	0.413	1.62 (0.55–4.79)	0.378

*P *values less than 0.05 were considered statistically significant

*H. pyloriHelicobacter pylori*, *BMI* body mass index, *OR* odd ratio, *CI* confidence interval

^a^%s may not add up to 100 as one patient be diagnosed with more than one Gastrointestinal sign and symptoms

The pathology report indicated that 109 (46.8%) and 124 (53.2%) patients were positive and negative for *H. pylori*, respectively. The results also showed a significant difference between the two groups in terms of mucosal erosions in the stomach and duodenum, as well as normal endoscopic findings (*P* = 0.001) (Table 3).

**Table 3:** Comparison of endoscopic findings by participants with positive and negative *H. pylori*

Endoscopic findings	Total N = 233 n (%)	*H. pylori* positive N = 109 n (%)^a^	*H. pylori* negative N = 124 n (%)	*P* value
Gastric ulcer	18 (7.7)	8 (44.4)	10 (55.6)	0.5
Duodenal ulcer	6 (2.5)	1 (16.7)	5 (83.3)	0.1
Mucosal erosions in the stomach	98 (42.0)	71 (42.4)	27 (27.6)	0.001
Mucosal erosions in the duodenum	68 (29.1)	55 (80.9)	13 (19.1)	0.001
Antral nodularity	13 (5.5)	3 (23.1)	10 (76.9)	0.06
Body nodularity	20 (8.5)	13 (65.0)	7 (35.0)	0.07
Duodenal nodularity	2 (0.01)	1 (50.0)	1 (50.0)	0.7
Normal	94 (40.3)	25 (26.6)	69 (73.4)	0.001

*P* values less than 0.05 were considered statistically significant

Table shows the various endoscopy findings. Ninety four patients (40.3%) had normal endoscopy results. Significantly more patients with *H. pylori* positive had mucosal erosions in the stomach, and duodenum

*H. pyloriHelicobacter pylori*

^a^%s may not add up to 100 as one patient be diagnosed with more than one endoscopic finding

The frequency of the patients’ pathological findings is shown in Table 4. The results showed a significant difference between the two groups regarding normal gastric glands and intestinal metaplasia (*P* = 0.001).

**Table 4:** Comparison of pathological findings by participants with *H. pylori* positive and negative

Pathological findings	Total N = 233 n (%)	*H. pylori* positive N = 109 n (%)^a^	*H. pylori* negative N = 124 n (%)	*P* value
Neutrophil infiltration	124 (53.2)	94 (75.8)	30 (24.2)	0.001
Abscess + mucin decrease	1 (0.04)	1 (100.0)	0 (0.0)	0.4
Normal gastric glands	80 (34.3)	2 (2.5)	78 (98.5)	0.001
Complete intestinal metaplasia	12 (5.1)	12 (100.0)	0 (0.0)	0.001
Incomplete intestinal metaplasia	28 (12.1)	27 (96.4)	1 (3.6)	0.001
Atrophy	84 (36.0)	44 (52.4)	40 (47.6)	0.1
High grade dysplasia	1 (0.04)	1 (100.0)	0 (0.0)	0.4

*P* values less than 0.05 were considered statistically significant

Table shows the various pathologic findings. Significantly more patients with *H. pylori* positive had neutrophil infiltration, complete intestinal metaplasia, and incomplete intestinal metaplasia compared with patients without *H. pylori*

*H. pyloriHelicobacter pylori*

^a^%s may not add up to 100 as one patient be diagnosed with more than one Pathological finding

## Discussion

In this study, 233 patients with gastrointestinal diseases were first examined for *H. pylori* infection, and then, they were divided into *H. pylori*-positive and *H. pylori*-negative groups. Analysis of demographic data showed that the rate of *H. pylori* infection was significantly higher in married people, patients with more than three family members, and patients with a history of cancer. Also, the incidence of gastrointestinal symptoms, including epigastric pain, weight loss, and loss of appetite, was significantly higher in *H. pylori*-positive patients as compared to *H. pylori*-negative patients.

Various studies have suggested that factors, such as age, congestion, high number of family members, and poor health, especially in childhood, are the main causes of infection with *H. pylori* [18–20]. Age is one of the most important factors in the development of *H. pylori* infection. Many recent studies have indicated a significant association between this factor and the incidence of *H. pylori* infection [21]. In a study by Rowland et al. [19], young children before the age of 3 years were at risk of *H. pylori* infection, whereas the risk of infection was very low after 5 years of age. In the present study, most people (59.5%) with *H. pylori* were in the age group of 35–45 years [19]. Similar to our findings, Toyoshima et al. [22] reported that patients in the age range of 40–59 years had significantly higher serum antibody titers for *H. pylori* as compared to other age groups.

In the present study, the incidence of *H. pylori* infection was not significantly different between males and females. The results of multiple studies are consistent with our findings, which showed that *H. pylori* infection is not sex-dependent [23].

While smoking consumption is well-known risk factors for the incidence of *H. pylori* infection, no significant difference was observed in the present study. Our results are similar to previous studies [24–27].

Also, the number of family members is one of the factors evaluated in the present study; however, no significant relationship was found between this factor and the incidence of *H. pylori* infection. On the other hand, many studies have reported inconsistent results. It is hypothesized that with an increase in the number of family members, the amount of contact with the outside environment increases, contact between people increases indoors, and therefore, the incidence of infection may increase [28].

Education is one of the factors that is inversely related to the incidence of *H. pylori* infection; in other words, people with higher education are less likely to have this type of disease [29]. Several previous studies have found the relation between lower education and a high prevalence of *H. pylori* [30–35]. However, this study showed that the infection rate of *H*. *pylori* among illiterate individuals was significantly lower than that in literate participants*. A* small sample size can also lead to this result.

In our study, unemployed participants were found to be infected with *H. pylori* more often than other participants. Several studies have analyzed and confirmed the relationship between socioeconomic status and frequency of *H. pylori* infection. [36, 37]. However, we were not able to confirm this, because in our study, the income of the participants was not clear.

Based on the present results, gastric and duodenal mucosal erosions were significantly more common in *H. pylori*-positive patients as compared to *H. pylori-*negative patients. Similar to our findings, a study by Lehmann et al. [38] showed that the rate of *H. pylori* infection in patients with gastric erosion was significantly higher than those negative for *H. pylori* (*P* < 0.01). Overall, gastric mucosal erosive changes may be acute or chronic. Erosive changes seem to be common in patients with peptic ulcer and hyperacidity and may be a manifestation of *H. pylori*-induced peptic ulcer in many patients. Notwithstanding, *H. pylori* is considered to be the primary cause of most types of gastritis, although its role as a causative agent in gastric erosions is unclear [39].

In this study, endoscopy showed that there was no difference between *H*. *pylori*-*negative* and *H*. *pylori*-*positive* in terms of gastric and duodenal ulcers. Previous study has revealed that more than 90% of duodenal ulcers and 60% of gastric ulcers are associated with *H. pylori* infection [40]. On the other hand, the incidence of peptic ulcer and gastritis is increasing in patients who are susceptible to *H. pylori* [41, 42]. However, the results of some studies are inconsistent with these findings [13, 42–44].

Comparison of pathological findings in patients with and without *H. pylori* infection showed that neutrophil infiltration was the most common finding, observed in 124 (53.2%) patients [45]. The results of this study revealed that the incidence and severity of neutrophil infiltration in to the mucosal layer and muscularis propria, normal gastric glands, complete intestinal metaplasia, and incomplete intestinal metaplasia were significantly higher in *H. pylori*-positive patients. In the current study, 75.8% of patients with *H. pylori* showed signs of neutrophil infiltration in the mucosal layer and muscularis propria. Besides, incomplete intestinal metaplasia and complete intestinal metaplasia were reported in 100% and 96.4% of cases, respectively. Intestinal metaplasia is characterized by the loss of mucus-secreting capillaries and their replacement by goblet cells in the epithelium, which manifests in three forms; the third form is known as incomplete intestinal metaplasia.

In a similar study, Fontham et al. [46] indicated that 87% of patients with gastric cancer had intestinal metaplasia, and significant differences were found with normal controls. Moreover, Silva et al. [47] reported the highest incidence of *H. pylori* in areas with incomplete intestinal metaplasia; this type of metaplasia indicates long-term damage to the mucosa and leads to cancer. There are different reports on the prevalence of *H. pylori* infection in chronic gastritis and intestinal metaplasia, and many studies have attempted to find the link between direct or indirect effects of *H. pylori* and histological findings, especially dysplasia and gastric cancer.

In the present study on patients with and without *H. pylori* infection, gastric atrophy was reported in 52.4% and 47.6% of patients, respectively; this difference was not statistically significant*.* In a similar study, Nordenstedt et al. [48] reported that 41.5% of patients with *H. pylori*-negative gastritis had atrophy. Overall, gastric mucosal atrophy is the endpoint of chronic processes, such as chronic gastritis associated with *H. pylori* infection, other unidentified environmental factors, and autoimmunity directed against gastric glandular cells [49]. Atrophic gastritis is mostly caused by *H*. *pylori* infection. Therefore, some people classified as *H*. *pylori*-negative were previously infected with this bacterium. However, we cannot determine whether atrophic gastritis resulted from *H*. *pylori* infection or autoimmunity. It may be associated with long-term *H. pylori* infection and an autoimmune process that progressively destroys the oxyntic mucosa [50].

## Limitation

This study has some limitations. The sample undertaken in a single medical center, Guilan Gastroenterology and Liver Clinic, which may not be a general representation of Iran although the patients came from a variety of socioeconomic backgrounds. In addition, the sample size was small for assessing some risk factors. Another limitation of this study is that we lacked information on personal income or monthly family income.

## Conclusions

The results of this study indicated that the prevalence of *H. pylori* was related to the number of family members and marital status. Our findings also indicated that *H. pylori* infection was not sex-dependent and that alcohol consumption, smoking, and use of non-steroidal anti-inflammatory drugs (NSAIDs) did not increase the incidence of *H. pylori* infection. Since the role of *H. pylori* in the physiopathology of gastric ulcer is well understood, in this study, patients with *H. pylori* showed significant differences in endoscopic and pathological findings, especially regarding gastric and duodenal erosions in the presence of complete or incomplete intestinal metaplasia. Considering the important role of *H. pylori* in gastric ulcer and the possible role of other factors in its development, it is recommended to compare the clinical features, endoscopic findings, and pathology reports of patients with gastric ulcer who are positive or negative for *H. pylori* infection. Since in the present study, the relationship between the used drugs and gastric ulcer was not investigated, in future studies, it is recommended to compare this association between different drugs and gastric ulcer.

## Data Availability

The datasets obtained during this study will be available upon request to the corresponding author.

## Abbreviations

*H. pylori*: *Helicobacter pylori*
NSAID: Non-steroidal anti-inflammatory drug
MALT lymphoma: Gastric marginal zone lymphoma of mucosa-associated lymphoid tissue
IRIDA: Iron-refractory iron deficiency anemia
BMI: Body mass index
RUT: Rapid urease test
OR: Odd ratio
CI: Confidence interval
